# Captain Cook


**Published:** 1890-11-29

**Authors:** 


### Turning Over New Leaves.

### Captain Cook.*

"The world knows nothing of its greatest men," says the sententious poet, and even when it thinks it knows, when it is familiar with their names, and has some idea of what they have done for it, the knowledge is so vague and inaccurate that perhaps a little conscious ignorance would be better. For example, we all know everything about Captain Cook. That is, we know that he lived some time in the last century, and circumnavigated the globe, and was killed by savages somewhere in the South Seas; but of the man himself "in his habit as he lived," we know almost nothing. Therefore we are the more indebted to Mr. Walter Besant for having filled up the hiatus in our minds by his contribution to the "English Men of Action" series. It is true that Mr. Besant's method might not commend itself to many estimable members of the great Dryasdust family, for he extracts some incidents from a volume which he calls "The Book of Things Forgotten." Mr. Besant has often given us extracts from this book before, and called them novels or stories, but this does not in the least depreciate the testimony he has drawn from it regarding Captain Cook. All wise people know that fiction—good fiction—is very much truer than fact, being indeed inferences drawn from facts by men who know the workings of human nature—the Cuviers of psychology. It would be unjust, however, to insinuate that Mr. Besant has merely drawn upon his imagination. He has investigated all available sources of information, and brought clearly before us the career of the hind's son, the grocer's apprentice, who became a captain in the Royal Navy—which was doubtless what impressed most deeply those who had known Cook in his youth—and who, as Mr. Besant truly says, gave to Great Britain the Greater Britain—Australia.

James Cook was born at Marton, a village in Yorkshire, on October 27th, 1728. His father is said to have been Scotch, and to have migrated from the village of Ednam, in Roxburghshire. His mother was a Yorkshire woman, so from both sides Cook inherited the firmness of purpose, the taciturnity and self-reliance that mark the north country folks. When he was eight the family removed to Great Ayton, and when he was thirteen he was apprenticed to a shopkeeper—grocer, draper, and general dealer—at a village called The Staithes, and from this occupation he ran away to sea, on, according to Mr. Besant, July 5th, 1742. Tradition says that he stole a shilling from his master's till. Even if this be true, as it may be, it does not wholly do away with James Cook's claim to be regarded as a hero. His seafaring life began on board a collier trading between Whitby and London. He made one or two voyages to Norway, but for thirteen years, by the end of which time he had risen to be mate, his life was mainly spent in coasters devoted to the coal trade. In 1756, war having been declared between England and France he volunteered for the Royal Navy. Here it was found that he understood the art of navigation, and within a year he was promoted to the rank of master. Immediately after this promotion he was ordered to take soundings of the St. Lawrence in front of Quebec, then occupied by the French. Having performed this difficult and dangerous service admirably he was assigned various tasks of a similar nature, and explored a great part of the almost unknown island of Newfoundland. In 1762 he married, but was allowed to remain at home only four months, when he was recalled to his American duties, which ended in 1767.

It was on August 26th, 1768, that the *Endeavour*, with Cook, now promoted to the rank of lieutenant, in command, sailed for the Pacific, with the special intention of observing a transit of Venus from the newly-discovered island of Otaheite. This was done, and more, for Cook spent six months in exploring the coast of New Zealand, and sailed along the coast of Australia for two thousand miles. In 1771 he returned to England, where he was promoted to the rank of commander. He had hoped to be made post-captain, but such an honour was held to be too high for him. But he was offered the command of a second expedition to the South Seas, with a view to discovering the great Antartic continent which formed the Hesperides of that age. In the last voyage many of Cook's men had died of scurvy; nearly all had suffered from it; and therefore among the stores he had put all manner of anti-scorbutics. The record of the next voyage is the story not only of struggles with wind and waves, with savages and with the impenetrable ice around the southern pole, but with that more deadly and constant foe, the scurvy. The disease still appeared, but that Cook's precautions—his sour-krout and lemons, his spruce tea, portable broth, mustard and marmalade—had been successful appears from the fact that only four men died during the three years' voyage, three by accident, and one, we are told, "by a disease which would, perhaps, have brought him to the grave much sooner had he continued in England."

After this voyage Cook was made a post-captain, and also a captain in Greenwich Hospital, so that his old age was provided for. What his wife thought of these voyages, in spite of the fame and promotion they brought, may be inferred from the fact that Cook did not tell her when he was offered the command of another expedition, and broke the news only when all was decided. We all know the end of that voyage —how Cook was murdered on the shore of Owhyhee, or Hawaii, in sight of his men. How far the murder was unprovoked it is difficult to say. We have not an account of it from the Owhyhee point of view, and Cook was always a stern man, and in this voyage especially had shown himself severe in his dealings with the natives, punishing petty thefts with mutilation, and seizing the sacred persons of kings and priests without the smallest compunction. It was in the attempt to make a monarch prisoner that he was killed by people who feared their master's life was in danger. There was something to be said on their behalf. But they deprived England of one of her greatest navigators; and Cook was not merely a navigator. He was one of the first to give serious attention to the health and comfort of men on board ship. A silent man and a stern commander, he was yet a man who looked after his crew. His journals record his satisfaction when the remedies he had taken with him proved efficacious in repressing the scurvy. He made spruce tea for his men, he boiled celery and scurvy grass with their peas; he made a sort of beer from sugar cane and administered it to them. He did not fidget and fume about the discomforts inevitable to going down to the sea in ships in the eighteenth century. When an inconvenience could not be cured it was endured in silence; but if it was remediable, Captain Cook would do his best to remove it. It is well that in these days of talk and introspection the story of this strong, quiet, unpretentious man of action should be written, and doubly well that it should be written by Mr. Besant in such fascinating style that he who reads the book, be he boy or man, cannot lay it down till it is finished.

* "Captain Cook," by Walter Besant. "English Men of Action" Series. (London, Messrs. Macmillan and Co.)

---

The guinea-pig is really to be pitied. In the nursery he is a subject of jokes, owing to his tail being so conspicuous by its absence; but, still worse, his vocation in life seems to be to minister to the insatiable desires of the vivisectionist. He is at present in great demand for the manufacture of lymph for inoculating consumptive patients.

